# Generation of Bioactive Stem Cell-Derived Secretome in 3D Bioreactor System: Towards Cell-Free Therapy in Veterinary Medicine

**DOI:** 10.3390/biom16010002

**Published:** 2025-12-19

**Authors:** Věra Daňková, Andrea Exnerová, Hana Vágnerová, Vojtěch Pavlík, Kristina Nešporová

**Affiliations:** 1 Contipro a.s., 56102 Dolní Dobrouč, Czech Republic; vera.dankova@contipro.com (V.D.); andrea.exnerova@contipro.com (A.E.); hana.vagnerova@contipro.com (H.V.);; 2 Department of Biochemistry, Faculty of Science, Masaryk University, 61137 Brno, Czech Republic

**Keywords:** canine adipose-derived mesenchymal stem cells, 3D bioreactor culture, microcarriers, hypoxia, platelet lysate, secretome, regenerative medicine, veterinary medicine

## Abstract

Canine adipose-derived mesenchymal stem cells (cASC) are promising for regenerative veterinary medicine due to their immunomodulatory and reparative capacities. Three-dimensional (3D) culturing provides a more physiologically relevant environment than conventional two-dimensional (2D) monolayers, enhancing paracrine activity and therapeutic potential of mesenchymal stem cells (MSC). This study investigates the production and biological characterization of cASC secretome generated under hypoxic conditions with platelet lysate (PLT) supplementation, either in a 2D culture or in a stirred-tank 3D culture. Secretomes obtained from 3D cultures were compared with those from 2D cultures prepared under identical hypoxic and PLT-supplemented conditions. Quantitative analyses revealed enhanced secretion of key factors, including monocyte chemoattractant protein-1 (MCP-1) and vascular endothelial growth factor (VEGF), in 3D-derived secretomes. Functional in vitro assays demonstrated superior anti-inflammatory, pro-migratory, and antifibrotic effects of the 3D secretome, evidenced by nuclear factor kappa B (NF-κB) inhibition, increased fibroblast migration, and modulation of extracellular matrix gene expression. Additionally, the bioreactor system enabled consistent secretome production with reproducible biological activity. These findings indicate that 3D bioreactor cultivation under hypoxia with PLT supplementation can generate a biologically active secretome from canine adipose-derived stem cells, providing a promising basis for further exploration in veterinary regenerative applications.

## 1. Introduction

Mesenchymalstem cells (MSC) are multipotent cells capable of self-renewal and differentiation into various mesenchymal lineages. They are commonly isolated from bone marrow, adipose tissue, umbilical cord, dental pulp and other tissues. Among these, adipose-derived mesenchymal stem cells (ASC) represent a well-characterized MSC subtype, offering easier accessibility and higher yield compared to other tissue sources. Beyond their regenerative capacity, MSC exert a wide range of immunomodulatory and trophic effects, primarily through the secretion of bioactive factors collectively referred to as the MSC secretome. This complex mixture, composed of cytokines, growth factors, extracellular vesicles, and other molecules, plays a key role in tissue repair, modulation of inflammation, and fibrosis attenuation [1,2,3]. In recent years, the cell-free therapeutic potential of the MSC secretome has attracted increasing attention as an alternative to direct cell therapy. Secretome-based therapies offer several advantages, including easier storage and handling, lower immunogenic risk, and reduced regulatory complexity [4]. While most studies have focused on human-derived MSC, there is a growing interest in applying this concept in veterinary medicine, particularly in canine patients, where regenerative therapies are increasingly sought for musculoskeletal, dermatological, and internal medicine conditions [5,6,7,8]. In both human and veterinary medicine, several pathological conditions are characterized by excessive inflammation, including nuclear factor kappa B (NF-κB) activation, impaired cell migration, and a pro-fibrotic microenvironment, which impair the natural healing process. For example, canine chronic enteropathies are associated with sustained NF-κB signalling in intestinal epithelial and immune cells, driving uncontrolled inflammation and barrier dysfunction [9]. Similarly, in canine masticatory muscle myositis, fibrotic tissue develops in the chronic phase following prolonged inflammation and muscle degeneration [10]. Together, these processes impair tissue repair and underscore the need for targeted therapeutic approaches in veterinary medicine.

Despite the promising therapeutic potential, the production of standardized and potent MSC secretome remains a challenge. Traditional 2D culture methods often lead to limited yields and variability in secretome composition. To overcome these limitations, 3D dynamic culture systems, such as bioreactors, have been explored to provide a more physiologically relevant environment that enhances MSC secretory activity. Bioreactor systems allow for controlled parameters, such as oxygen tension, pH, nutrient supply, and shear stress, enabling scalable production, and improved reproducibility [11,12]. However, their application in the context of canine MSC has not been extensively studied. In parallel, platelet lysate (PLT) has been increasingly used in regenerative medicine due to its safety, accessibility, growth factor content, and its ability to reduce the risk of xenogeneic contaminants and batch-to-batch variability [13,14,15,16]. It has been shown to be a safe and effective alternative to fetal bovine serum (FBS) for adipose-derived mesenchymal stem cells (ASC) culture, supporting their proliferation and maintaining their regenerative properties [17]. Moreover, hypoxic culture conditions have been shown to mimic the native MSC niche, enhancing cell viability and promoting the secretion of pro-regenerative factors, which may further improve the therapeutic quality of the secretome [18,19].

While secretome research in human regenerative medicine has advanced considerably, studies in the veterinary field are still limited. The combined impact of 3D dynamic culture in a stirred-tank bioreactor, hypoxic conditions, and supplementation with platelet lysate on the quality and function of canine adipose-derived stem cell secretome has not been systematically explored. This study addresses this gap by preparing and functionally characterizing cASC secretome under these optimized culture conditions. The biological activity of the secretome was evaluated using multiple in vitro assays, including enzyme-linked immuno sorbent assay (ELISA)-based quantification of pro-regenerative cytokines, a scratch assay for fibroblast migration, an NF-κB reporter assay to assess anti-inflammatory effects, and a fibrosis model analysing the expression of extracellular matrix (ECM)-related genes, (COL1A1) and hyaluronan synthase 2 (HAS2). For comparative analysis, secretomes obtained from conventional 2D cultures prepared under the same hypoxic and PLT-supplemented conditions were also included. By integrating these approaches, this work provides new insights into how this culture strategy enhances the regenerative, anti-inflammatory, and anti-fibrotic potential of the secretome, offering novel perspectives for secretome-based strategies in veterinary regenerative medicine.

## 2. Materials and Methods

### 2.1. Cell Isolation and Culture

Adipose-derived mesenchymal stem cells were isolated from visceral adipose tissue collected opportunistically during ovariohysterectomy procedures in female dogs, with prior consent from the owner. The details on sex, breed, and age of the animals for each batch are provided in Table 1. As the tissue was considered biomedical waste collected during standard clinical procedures, the protocol was exempt from Institutional Animal Care and Use Committee (IACUC) approval. Approximately 3 g of adipose tissue was washed in phosphate-buffered saline (PBS), with 5% penicillin/streptomycin (Diagnovum Germany GmbH, Greifswald, Germany.), minced, and digested in 2% collagenase type IV (MEM-α, 2% penicillin/streptomycin) at 37 °C, 200 RPM for 60 min. The digested suspension was filtered through a 40 µm mesh and enzyme activity was quenched with culture medium (MEM-α, 10% FBS, 1% L-glutamine, and 100 U/mL pen/strep). Cells were centrifuged (5 min, 1200 RPM), washed with PBS, and resuspended in fresh culture medium. Cells were then seeded into T-150 flasks (Techno Plastic Products AG, Trasadingen, Switzerland) and maintained at 37 °C in a humidified incubator with 5% CO_2_. The culture medium was replaced every three days. Cells were passaged upon reaching 80–90%. Experiments were performed using cells at passages p1-p4. Cell counts were obtained using a Casy Cell Counter and were either cryopreserved or used immediately.

### 2.2. Flow Cytometric Analysis of cASC

Flow cytometry was used to characterize cASC using antibodies against CD29 (PE, Invitrogen, Carlsbad, CA, USA, 12-0299-42), CD44 (PE, Invitrogen, 12-0441-82), CD90 (PE, Invitrogen, 12-5900-41), and CD45 (FITC, Invitrogen, MA1-80428). An unstained sample was included as a negative control. Data acquisition was performed using a NovoCyte flow cytometer (ACEA Biosciences Inc, San Diego, CA, USA) and analyzed using NovoExpress software, version 1.3.0.

### 2.3. Secretome Production Under 2D Conditions

Secretome was prepared from cASC obtained from three independent donors, similarly to the approach described in. cASC cultures were seeded at a density of 4 × 10^4^ cells/mL in T75 culture flasks containing 10 mL of medium; no microcarriers were used for 2D secretome production. After 4 days of cultivation, the culture medium was replaced with RPMI 1640 without L-glutamine and phenol red, supplemented with 2% human platelet lysate (PLT; PLTGold, SCM151, Sigma-Aldrich, Darmstadt, Germany, xeno-free, clinical-grade), 2 mM L-glutamine (Glentham Life Sciences, Corsham, UK), 1 mM sodium pyruvate, glucose, 1% penicillin/streptomycin. After 72 h under 5% hypoxia, the secretome was collected (10 mL), centrifuged (2500 g, 10 min, 4 °C) and filtered through a 0.22 µm syringe filter. The medium was aliquoted into 1 mL portions, labelled as SEC 2D, and stored at −80 °C for up to 1 month for further use.

### 2.4. Preparation of Microcarriers

The gelatin-based macroporous microcarriers Cultispher-S (Parcell Biolytica, Åstorp, Sweden, Cat. No. M9043) with density of 1.02–1.04 g.cm^3^ and particle size of 130–380 μm were prepared for cell culture according to the manufacturer’s specifications. Initially, they were hydrated overnight using 50 µL of PBS without Ca^2+^ and Mg^2+^ per 1 mg of microcarriers. The microcarriers were then washed twice with fresh PBS (without Ca^2+^ and Mg^2+^) and subsequently sterilized by autoclaving (121 °C, 15 min). After sterilization, the microcarriers were washed twice with culture medium. Microcarriers were prepared prior to immediate use.

### 2.5. Cultivation of cASC on Microcarriers in 24-Well Plates

cASC were seeded into non-adhesive 24-well plates (Biofil, Madrid, Spain). Each well contained 2 mg/mL of Cultispher-S. The experiment involved three different cell seeding densities: 0.5 × 10^5^, 0.75 × 10^5^, and 1 × 10^5^ cells/mL in a total volume of 1 mL of culture medium. The cASC were incubated for 6 days on an orbital shaker at 40 RPM in a CO_2_ incubator. The culture medium was replaced after 3 days, with additional microcarriers added at a concentration of 2 mg/mL. Each condition was performed in triplicate and repeated in three independent biological replicates.

### 2.6. Cultivation of cASC in Stirred-Tank Bioreactor

cASC were cultured in a small-scale stirred-tank bioreactor (MiniBio, Applikon, Sweden) using 1 mg/mL Cultispher S microcarriers (Cat. No. M9073, Sigma-Aldrich, Stockholm, Sweden) in a working volume of 60 mL of culture medium (MEMα, 10% FBS, 2 mM L-glutamine, 1% penicillin/streptomycin). Prior to use, the bioreactor vessel was coated with Sigmacote (Sigma-Aldrich, St. Louis, MO, USA), autoclaved (121 °C, 15 min), and all sensors (temperature, pH, dissolved oxygen) were calibrated according to manufacturer’s instructions. Cells were inoculated at a density of 4 × 10^4^ cells/mL and cultured for 9 days. The pH (7.2–7.4) was maintained via CO_2_ headspace control, and dissolved oxygen was set to 20% air saturation by flushing the headspace with the mixture of air and N_2_. Temperature was maintained using a heated jacket. Agitation was set at 85 RPM and increased daily to 120 RPM for 10 min to prevent microcarrier sedimentation. Aeration was provided via headspace (overlay) gassing. Cultures were maintained under normoxic (21% O_2_) and hypoxic (5% O_2_) conditions. Complete medium changes were performed on days 3, 6, and 8. Sampling was performed under increased agitation (120 RPM) to ensure homogeneity. A 2 mL sample was withdrawn using a syringe for qualitative and quantitative assessment via fluorescence microscopy and MTT assay. At the end of cultivation, the culture supernatant was first removed, and cells were enzymatically detached from the microcarriers. For cell release from Cultispher S, a combination of trypsin/EDTA 5× (Cat. No. XC-T1717, Biosera, Nuaille, France) and dispase 7.2 U/mL (Cat. No. 445992, Gibco, Tokyo, Japan) was used in a 1:1 ratio. The total volume of the enzymatic solution was adjusted to 60 µL per 2 mg of Cultispher S.

### 2.7. Secretome Production in a Bioreactor

To produce the cASC secretome in a bioreactor, culture parameters were optimized. Cells were seeded at a density of 4 × 10^4^ cells/mL in 60 mL of culture medium. After adding RPMI medium with 2% PLT supplementation, microcarrier aggregation was observed under the standard 3D culture conditions. This aggregation was considered undesirable and was resolved by increasing the agitation speed to 110 RPM on day 2, preventing further aggregation. Additionally, the cultivation period was reduced to 4 days. After 4 days, culture was washed twice with 50 mL of PBS., and the culture medium (αMEM supplemented with 10% FBS, 2 mM L-glutamine, 1% penicillin/streptomycin) was replaced with 60 mL of RPMI medium without phenol red and glutamine supplemented with 2% PLT, 0.013 M D-glucose, 2 mM L-glutamine, and 1 mM sodium pyruvate. The agitation speed was adjusted to 110 RPM for an additional 72 h. Following this period, stirring was stopped, the bioreactor was disconnected and transferred to a laminar flow hood, where the secretome was carefully collected into 50 mL tubes. The medium was centrifuged (4 °C, 2500 g, 10 min) and the supernatant was filtered through a 0.22 µm syringe filter to obtain non-concentrated secretome, labelled as SEC 3D. To concentrate the secretome, samples were transferred to centrifugal filter units (Amicon, Burlington, MA, USA; 3 kDa) and processed by centrifugation at 4 °C, 4000 g for 45 min to obtain a 5× concentrated secretome, labelled as SEC 3D 5×. Samples were aliquoted and stored at −80 °C for further biological analyses, with a maximum storage time of up to 1 month To reduce variability and improve the reproducibility of the biological effects of the final product, secretome preparations were pooled from 3 different cASC batches, similarly to the approach used by [20].

### 2.8. Quantification of Pro-Regenerative Factors in the Secretome

The concentrations of selected pro-regenerative factors in cASC secretome were determined using ELISA. Commercial canine-specific ELISA kits were used to quantify VEGF-A and MCP-1 (RayBiotech, Peachtree Corners, GA, USA; Cat. No. ELC-VEGF and ELC-MCP1, respectively), according to the manufacturer’s protocols. Absorbance was measured at 450 nm using an Ensight microplate reader (PerkinElmer, Waltham, MA, USA).

### 2.9. Macrophage Differentiation and Secretome Treatment

U937 monocytes were seeded at a density of 8 × 10^4^ cells/cm^2^ in 1 mL of RPMI-1640 *w*/*o* phenol red, *w*/*o* glutamine with 10% FBS, 2 mM L-glutamine, 1% penicillin/streptomycin, 1 mM sodium pyruvate and 50 ng/mL phorbol 12-myristate 13-acetate (PMA, Cat. No. P1585, Sigma-Aldrich, St. Louis, MO, USA) to induce differentiation. After 96 h of incubation, the medium was replaced with fresh RPMI-1640 with 10% FBS, and cells were incubated for an additional 24 h to allow maturation into adherent macrophage-like cells. Differentiated macrophages were then stimulated with 100 ng/mL lipopolysaccharide (LPS, Cat. No. L9143–25 MG, Sigma-Aldrich, St. Louis, MO, USA) combined with cASC secretome samples diluted 1:1 with culture medium for 48 h. After incubation, cells were lysed, total RNA was isolated, and TNF-α mRNA expression was quantified using qPCR with a TaqMan gene expression assay.

### 2.10. NF-κB Reporter Assay

Murine macrophage RAW264.7 cells were transfected with the pNL3.2.NF-κB-RE[NlucP/NF-κB-RE/Hygro] vector (Cat. No. N1111, Promega, Madison, WI, USA), which encodes NanoLuc^®^ luciferase under the control of NF-κB response elements. Transfection was performed using FuGENE^®^ HD Transfection Reagent (Cat. No. E2311, Promega, Madison, WI, USA) according to the manufacturer’s protocol. Prior to transfection, a hygromycin B killing curve was established to determine the minimal antibiotic concentration required for complete elimination of non-transfected cells. Following transfection, cells were cultured in medium containing hygromycin B at the predetermined selection concentration until a stable population of resistant cells expressing the reporter construct was obtained.

Prepared reporter cell line was used to assess the effect of cASC secretome on LPS-induced NF-κB activation. Cells were seeded into 24-well plates at a density of 5 × 10^5^ cells/well in low glucose Dulbecco’s Modified Eagle Medium (Gibco (Thermo Fisher Scientific), Grand Island, NY, USA) supplemented with 10% FBS, 1% penicillin/streptomycin, 2 mM L-glutamine, and incubated for 24 h at 37 °C with 5% CO_2_. The next day, cells were treated with cASC secretome for 24 h, followed by stimulation with lipopolysaccharide (LPS, PSAE, 10 ng/mL). Controls included unstimulated cells without LPS (negative control, NC) and cells stimulated with LPS alone (positive control, LPS). After 1 h of incubation, NF-κB reporter activity was determined by measuring luminescence using the Nano-Glo^®^ Luciferase Assay (Promega, Madison, WI, USA) according to the manufacturer’s instructions. Luminescence was measured using an Ensight microplate reader (PerkinElmer, Waltham, MA, USA).

### 2.11. Scratch Assay for Analysis of Cell Migration

The scratch assay was used to evaluate the effects of secretome on cell migration. NIH 3T3 fibroblasts (ATCC, Manassas, VA, USA) were seeded into 96-well ImageLock plates (Sartorius, Göttingen, Germany) at a density of 4.5 × 10^3^ cells/well in 200 μL of medium DMEM supplemented with 10% FBS, 1% penicillin/streptomycin, 2 mM L-glutamine, 20 mM D-glucose. The following day, cells were starved in serum-free medium for 24 h to minimize proliferation. After starvation, medium was removed, cells were washed with PBS (100 µL), and uniform scratches were made using the WoundMaker tool (Sartorius, Göttingen, Germany). Cells were gently washed with PBS, followed by the addition of 100 µL of serum-free medium. cASC secretomes (200 µL) were then added to test their effects on cell migration. Negative control (NC; 0% FBS) and positive control (PC; 10% FBS) were included to compare the effects on cell migration. Cell migration was monitored for 24 h using the IncuCyte S3 Live-Cell Analysis System (Sartorius, Göttingen, Germany) and quantified based on cell confluence within the wound area using IncuCyte software (version 2019B).

### 2.12. TGF-β1-Induced Fibroblast Activation Assay

Fibroblast-to-myofibroblast differentiation was induced using the cytokine transforming growth factor beta-1 (TGF-β1). NIH-3T3 mouse embryonic fibroblasts (ATCC, Manassas, VA, USA) were seeded into 6-well plates at a density of 2 × 10^5^ cells/well in DMEM (Biosera, Nuaille, France), supplemented with 10% FBS, 1% penicillin/, streptomycin, and 2 mM L-glutamine. On the following day, the growth medium was removed, cells were washed with PBS, and serum-free medium was added. On day three, cells were treated with serum-containing medium supplemented with 10 ng/mL TGF-β1 (Cat. No. ab50036, Abcam, Cambridge, UK) along with the tested cASC secretome. After 48 h of stimulation, expression levels of fibrosis-related markers COL1A1 (Assay ID Mm00801666_g1, Thermo Fisher Scinetific, Waltham, MA, USA) and HAS2 (Assay ID Mm00515089_m1, Thermo Fisher Scinetific, Waltham, MA, USA) were analyzed by qPCR.

### 2.13. Real-Time PCR Analysis

Total RNA was isolated using the RNeasy Mini Kit (Cat. No. 74116, QIAGEN, Hilden, Germany) and the QIAcube automated system (QIAGEN). Complementary DNA (cDNA) synthesis was performed from 1000 ng of RNA using the High-Capacity cDNA Reverse Transcription Kit (Thermo Fisher Scientific, Waltham, MA, USA) according to the manufacturer’s instructions and gene expression was quantified by qRT-PCR with TaqMan Fast Advanced Master Mix (Applied Biosystems, Foster City, CA, USA). qRT-PCR was carried out using TaqMan Gene Expression Assays using a QuantStudio system (Thermo Fisher Scientific, Waltham, MA, USA). Ct values were normalized to GAPDH as an endogenous control and analyzed using the 2^−ΔΔCt^ method [21]. Specific TaqMan probes used are listed in the Materials and Methods sections describing the respective biological assays.

### 2.14. Cell Proliferation and Viability Assays on Microcarriers

Cell proliferation on microcarriers was assessed using calcein-AM and Hoechst 33342 staining. Calcein-AM (Cat. No. 206700, Sigma-Aldrich, St. Louis, MO, USA; 1 µg/mL) and Hoechst (Cat. No. H21486, Thermo Fisher Scientific, Waltham, MA, USA; 0.1 µg/mL) were diluted in PBS and applied to the samples (500 µL per well) followed by 15 min incubation at 37 °C and 5% CO_2_. Fluorescence imaging was performed using a Nikon Eclipse-Ti microscope (Nikon Corporation, Tokyo, Japan).

Additionally, cell viability was measured using the MTT assay. After removing supernatants, 400 µL of cASC medium (MEMα, 10% FBS, 2 mM L-glutamine, 1% penicillin/streptomycin) and 40 µL of MTT solution (5 mg/mL in PBS) were added. Plates were incubated for 2.5 h at 37 °C and 5% CO_2_. Due to the presence of microcarriers, only 200 µL of medium was removed to avoid disturbing the cells, after which 200 µL of solubilization solution (45:45:10 mixture of isopropanol, DMSO, and Triton X-100) was added and the plates were incubated for an additional 2 h at room temperature with shaking. Absorbance was read at 470 and 690 nm.

### 2.15. Statistical Analysis

Data were analyzed using GraphPad Prism 10 software. Specific statistical tests applied for each experiment are indicated in the respective figure legends. Results are presented as mean ± standard deviation (SD) unless stated otherwise.

## 3. Results

The experimental workflow was designed to assess the effects of 3D dynamic culture, platelet lysate supplementation, and hypoxic preconditioning on the pro-regenerative secretome of cASC. Following cell isolation and characterization, preliminary experiments were conducted to optimize culture conditions on microcarriers, which were then applied in 3D bioreactor cultivation. The resulting secretome was subsequently collected for in vitro analysis.

### 3.1. Optimization of cASC Cultivation on Microcarriers in a 24-Well Plates

**Figure 1:**
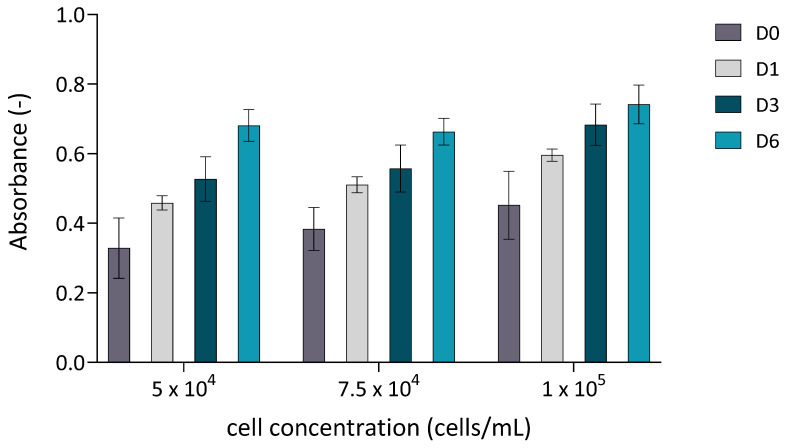
Cell proliferation quantitatively assessed using the MTT assay. cASC were cultured on Cultispher S microcarriers (2 mg/mL) at three different initial seeding densities (5 × 10^4^, 7.5 × 10^4^, and 1 × 10^5^ cells/in 24 wells. Absorbance was measured at four time points: D0 (2 h after seeding), D1, D3, and D6. Data are presented as mean ± standard deviation (*n* = 3).

To determine the optimal cell-to-microcarrier ratio, a standard screening analysis was performed in 24-well plates. The cultivation was carried out under dynamic conditions, as the plates were placed on an orbital shaker at 40 RPM. Cell proliferation was quantitatively assessed using the MTT assay at four time points: D0 (2 h after seeding), D1, D3 and D6. The results (Figure 1) revealed a time-dependent increase in absorbance for all tested cell concentrations, indicating progressive cell proliferation on the microcarriers. Despite slight differences in metabolic activity between the tested groups on day 6, the data suggest that lower MSC-to-microcarrier ratios may support more efficient growth. Based on these findings, and with the intention of reducing the initial number of cells while still achieving sufficient expansion, we selected a seeding density of 4 × 10^4^ cells for the subsequent bioreactor experiments. Moreover, this concentration aligns with previous findings [22,23], where lower MSC-to-microcarrier ratios were found to be optimal. To further enhance cell yield, an additional batch of microcarriers was introduced on day 3 of cultivation, a strategy previously reported to increase total cell output [24,25].

**Figure 2:**
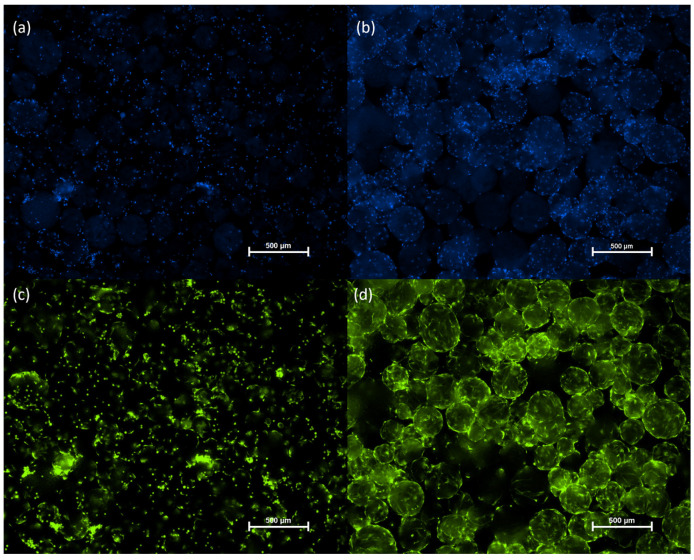
Microscopic images of cASC cultured on Cultispher S microcarriers (2 mg/mL) in 24-well plates. Cells were seeded at an initial density of 5 × 10^4^ per well in a 24-well plate placed on an orbital shaker (40 RPM) in an incubator (37 °C, 5% CO_2_). An additional 1 mg/mL of microcarriers was added on day 3 of cultivation. Hoechst staining on hour 4 (**a**) and day 6 (**b**). Calcein staining on hour 4 (**c**) and day 6 (**d**) of cultivation. Hoechst staining (blue), Calcein staining (green).

Cell adhesion and proliferation on the microcarriers were monitored via fluorescence microscopy using dual staining with Calcein AM and Hoechst 33342. Calcein AM selectively labeled viable cells with green fluorescence, while Hoechst 33342 stained all cell nuclei with blue fluorescence. Fluorescence imaging confirmed successful attachment of cASC to the microcarrier surface as early as 4 h after seeding (Figure 2a,c). After 6 days of cultivation, a substantial increase in cell density was observed (Figure 2b,d), indicative of active cASC proliferation on the microcarriers under the applied culture conditions.

These preliminary optimization experiments provide the basis for the 3D bioreactor cultivation described in Section 3.2.

### 3.2. Dynamic 3D Culture of cASC on Cultispher S Microcarriers Supports Cell Viability and Proliferation over Time

**Figure 3:**
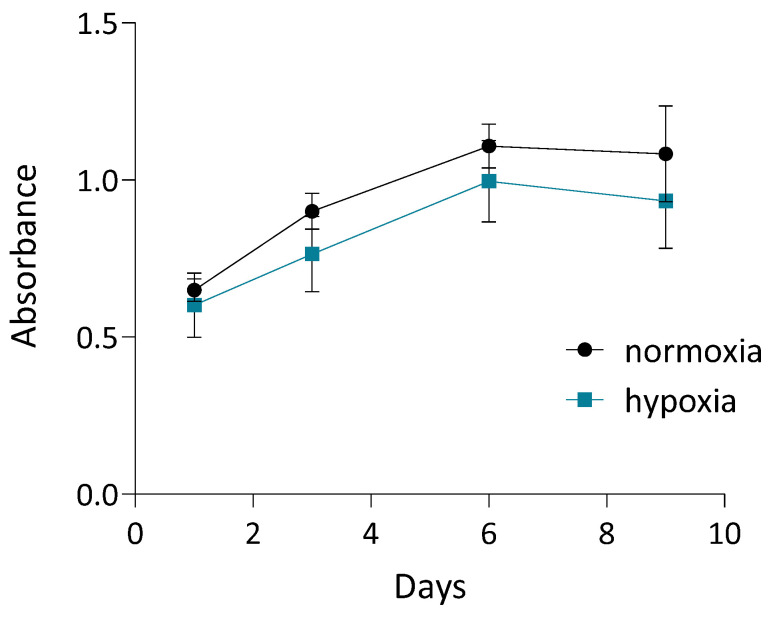
Quantification of cASC proliferation using MTT assay on days 1, 3, 6, and 9 of 3D dynamic culture under normoxic and hypoxic conditions. Values represent mean ± SD. Complete medium exchange was performed on days 3, 6, and 8.

To assess cell proliferation in 3D dynamic culture, cASC were seeded onto Cultispher S microcarriers, and metabolic activity was quantified using the MTT assay on days 1, 3, 6, and 9. As shown in Figure 3, cASC exhibited a progressive increase in metabolic activity over time, indicating sustained viability and active proliferation within the 3D culture system. This trend was observed under both normoxic and hypoxic conditions, although metabolic activity was slightly reduced in hypoxia throughout the culture period. Both conditions were tested initially to ensure hypoxia did not impair cell growth. Since no negative effects were observed, secretome preparation for subsequent tests was carried out under hypoxic conditions to better mimic physiological environments and are known to enhance the regenerative potential of the secretome.

**Figure 4:**
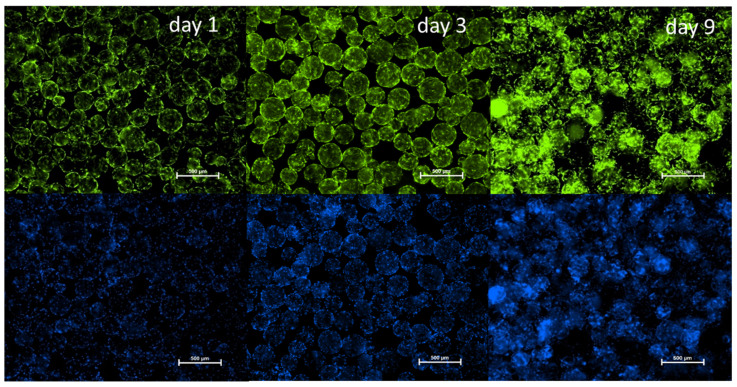
Fluorescence microscopy of cASC cultured on Cultispher S microcarriers on days 2, 3, and 9 under dynamic conditions. Viable cells stained green with Calcein-AM; nuclei stained blue with Hoechst 33342. Scale bars = 500 µm.

To complement these findings, fluorescence microscopy was used to visualize cell attachment and distribution on days 1, 3, and 9. Viable cells were stained with Calcein-AM (green), and nuclei were labeled with Hoechst 33342 (blue). As shown in Figure 4, cASC adhered to the microcarriers and began spreading by day 1. By day 3, an increase in cell density and spreading was evident. On day 9, the microcarrier surfaces were covered by a mostly continuous, although not fully uniform, single-cell layer of cASCs, indicating successful colonization and progressive growth. Therefore, Cultispher S-based 3D dynamic culture system effectively supports the proliferation and viability of cASC over time.

**Figure 5:**
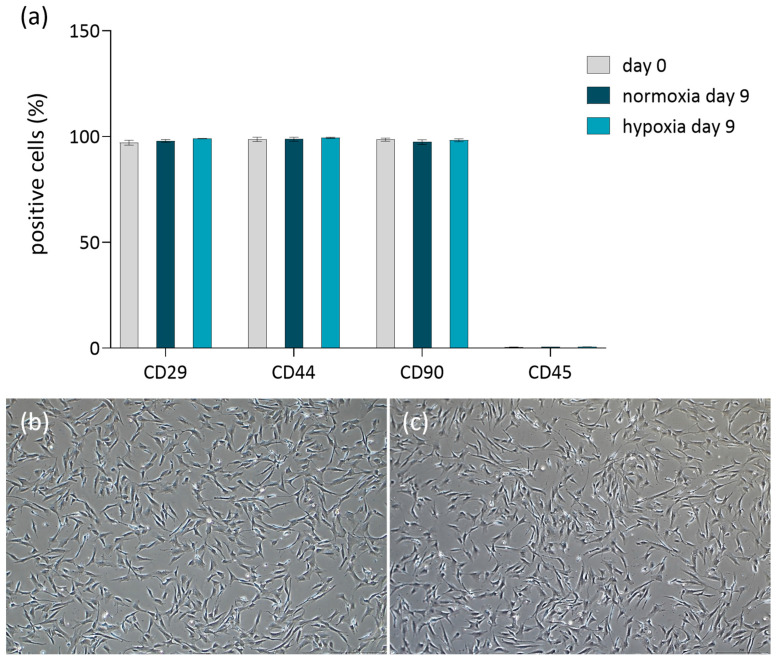
Immunophenotype and adherence capability of cASC following 9 day dynamic culture in the bioreactor. (**a**) Immunophenotypic analysis of cASC before culture (grey bars) and after culture under normoxic (dark turquoise) and hypoxic (light turquoise) conditions. (**b**,**c**) Morphological assessment of cASC adherence after bioreactor culture under normoxic (**b**) and hypoxic (**c**) conditions.

The presence of mesenchymal stem cell surface markers was successfully confirmed by flow cytometry analysis at the end of cultivation (day 9), see (Figure 5a). The immunophenotypic profile remained consistent before and after culture under both normoxia and hypoxia, with positive expression of CD29, CD44, and CD90, and absence of the hematopoietic marker CD45. These findings, including the characteristic adherence of the cells to plastic surfaces (Figure 5b,c), confirm the identity and typical behavior of MSCs according to the criteria of the International Society for Cellular Therapy [26], following the applied culture protocol.

### 3.3. 3D Culture Enhances Secretion of Pro-Regenerative Factors MCP-1 and VEGF

To optimize secretome composition and downstream applications, cASCs were cultured under hypoxic conditions (5% O_2_) and in medium supplemented with PLT. Secretomes were obtained from cells cultured either in 2D static monolayers on tissue culture flasks (SEC 2D) or in a 3D dynamic system (SEC 3D), with equal cell seeding density across both systems. Cells were first cultured for four days in FBS-containing medium to allow adhesion and proliferation. Subsequently, the medium was replaced with PLT-supplemented medium, and cells were cultured for an additional 72 h to enable accumulation of secreted factors. Hypoxic conditions were applied during this secretome collection phase in both systems. To allow for downstream functional assays in a dose-dependent manner, SEC 3D samples were subsequently concentrated (SEC 3D 5×).

**Figure 6:**
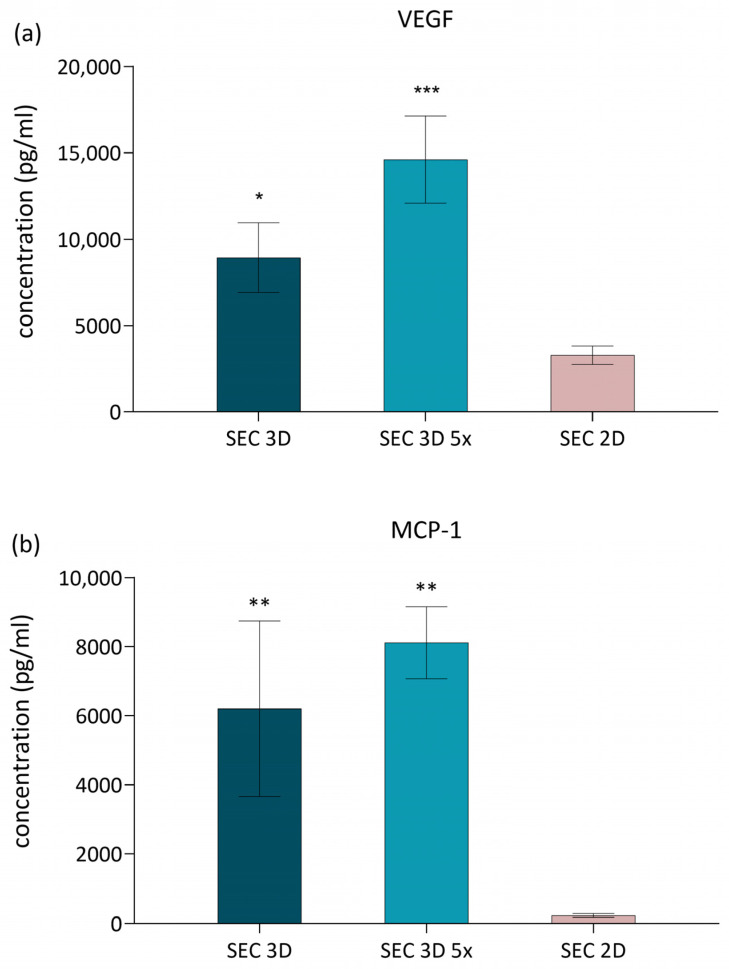
Effect of cASC-derived secretome on VEGF and MCP-1 secretion. (**a**) VEGF and (**b**) MCP-1 concentrations in cASC secretome from 3D dynamic (SEC 3D, SEC 3D 5×) and 2D static (SEC 2D) cultures under 5% O_2_ and PLT supplementation. Data are presented as mean ± SD (*n* = 3). Statistical significance was calculated in comparison to SEC 2D; * *p* < 0.05, ** *p* < 0.01, *** *p* < 0.001 one-way ANOVA followed by Dunnett’s post hoc test.

VEGF and MCP-1 were selected as representative factors because they are well-established markers of MSC pro-regenerative and angiogenic activity, widely reported in the literature [27,28,29]. To evaluate the effect of 3D dynamic culture on the secretion of pro-regenerative factors, MCP-1 and VEGF levels were quantified in SEC 2D and SEC 3D samples. VEGF concentrations were significantly elevated in both the SEC 3D and SEC 3D 5× compared to the 2D condition (Figure 6a). MCP-1 levels were also increased in SEC 3D and SEC 3D 5× relative to SEC 2D (Figure 6b). However, statistical significance was observed only in the concentrated 3D condition (SEC 3D 5×; *p* < 0.05), while the non-concentrated SEC 3D displayed a similar trend without reaching significance.

### 3.4. TNFα Suppression by cASC Secretome in Activated Macrophages

**Figure 7:**
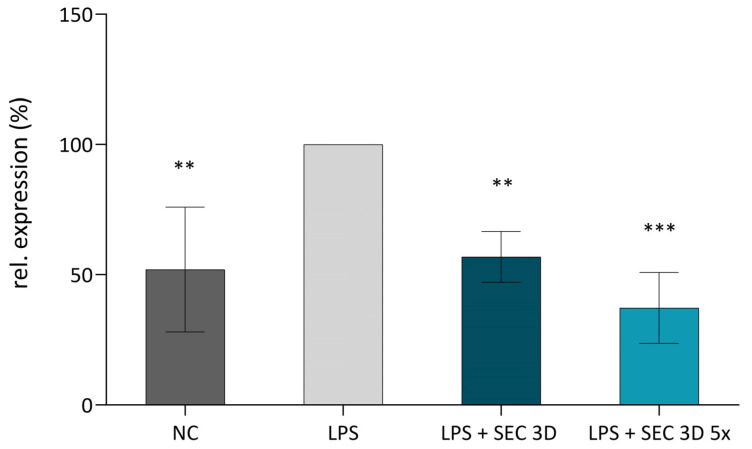
Effect of SEC 3D on TNFα expression in activated M1 macrophages. The U937 macrophages were activated using LPS at a concentration of 100 ng/mL. The experimental groups included a negative control (NC, non-activated macrophages without LPS), a positive control (LPS, macrophages + LPS), and macrophages treated with LPS in combination with SEC 3D (LPS + SEC 3D) and concentrated SEC 3D 5× (LPS + SEC 3D 5×). Data represent the means ± SD from at least four independent experiments and are shown relative to the positive control (LPS). Statistical significance was assessed using one-way ANOVA followed by Dunnett’s post hoc test: ** *p* < 0.01, *** *p* < 0.001.

TNFα is a key mediator of the inflammatory response, and its modulation is a critical target in anti-inflammatory strategies. Secretomes derived from 3D cultures may contain bioactive factors capable of attenuating inflammation. Therefore, we investigated their ability to reduce TNFα expression in LPS-stimulated U937 macrophages. TNFα expression in U937 macrophages was evaluated following treatment with secretomes derived from 3D cultures. The positive control (LPS, U937 macrophages stimulated with LPS) exhibited the highest level of TNFα expression, whereas the negative control (NC; macrophages without LPS stimulation) showed a markedly lower level of expression. Both the SEC 3D and its concentrated form SEC 3D 5× significantly reduced TNFα expression in LPS-stimulated macrophages compared to LPS-alone control (Figure 7).

### 3.5. 3D Culturing of cASC Enhances NF-κB Inhibitory Activity of the Secretome

**Figure 8:**
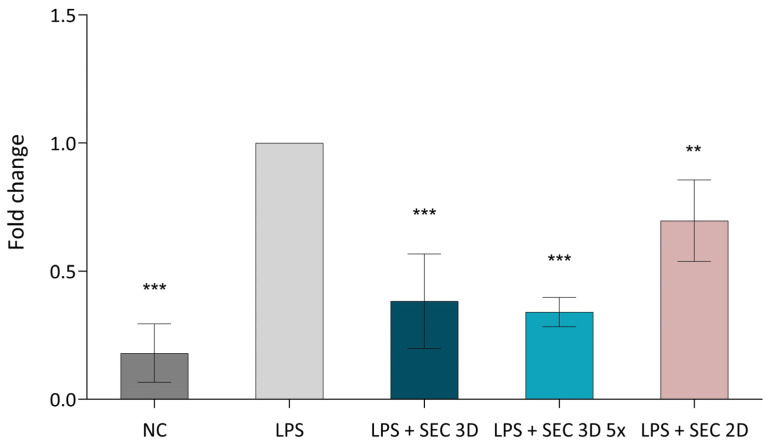
Effect of cASC secretome on NFκB signaling activity in RAW264.7 cells. The NFκB signaling pathway was activated using LPS at a concentration of 10 ng/mL, followed by treatment with SEC 3D, concentrated SEC 3D 5× or SEC 2D. Values represent the means from five independent experiments ± SD. Results are expressed as fold change relative to the positive control (LPS). The negative control (NC) corresponds to non-activated macrophages without LPS stimulation. Statistical significance was calculated in comparison to positive control (LPS) consisting of RAW264.7 macrophages stimulated with LPS. ** *p* < 0.01, *** *p* < 0.001, one-way ANOVA followed by Dunnett’s post hoc test.

NFκB expression in macrophages was assessed after treatment with SEC 3D and SEC 2D. The positive control (LPS; RAW264.7 macrophages + LPS) showed the highest activation, while the negative control (NC; macrophages without LPS) showed lower expression. SEC 3D and concentrated form (SEC 3D 5×) significantly reduced NF-κB activation to 38% and 34% of positive control, respectively. SEC 2D also reduced NFκB activation, but to a lesser extent (Figure 8).

### 3.6. Effect of cASC Secretome on the Fibrotic Phenotype Induced by TGF-β1 in 3T3 Cells

**Figure 9:**
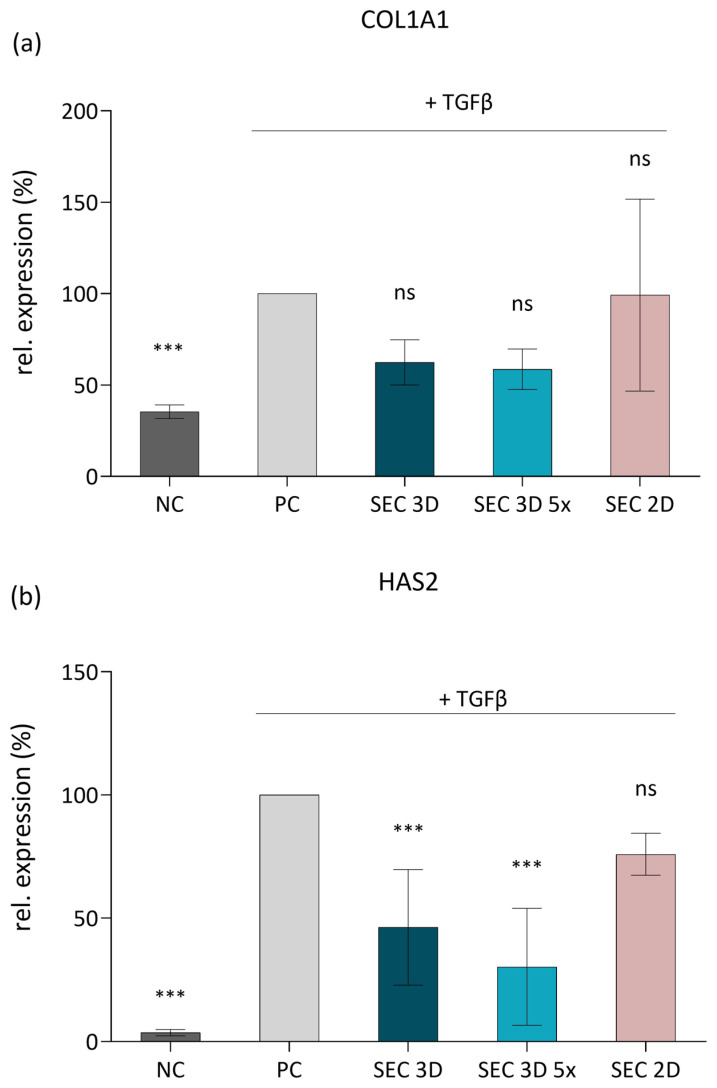
Effect of cASC secretome on ECM-related gene expression in TGF-β1-stimulated 3T3 fibroblasts. Expression levels of *COL1A1* (**a**) and *HAS2* (**b**) were determined by RT-qPCR after 48 h of stimulation with TGF-β1 and subsequent treatment with SEC 3D, concentrated SEC 3D 5×, or SEC 2D. Data are presented relative to the positive control (PC; 3T3 fibroblasts activated with TGF-β1). Bars represent mean values from at least free independent experiments ± SD. Statistical significance was calculated in comparison to PC; *** *p* ≤ 0.001, ns = not significant; one-way ANOVA followed by Dunnett’s post hoc test.

To assess the anti-fibrotic potential of cASC secretome, we utilized a model based on TGF-β1-stimulated NIH 3T3 fibroblasts. Stimulation of the cells with TGF-β1 (10 ng/mL) significantly upregulated their expression of *COL1A1* (encoding the α1 chain of type I collagen) and *HAS2* (encoding hyaluronan synthase 2), confirming induction of a fibrotic phenotype (Figure 9). Treatment with SEC 3D attenuated the expression of both *COL1A1* and *HAS2*. This inhibitory effect was more pronounced following concentration of the secretome, suggesting a dose-dependent response. In contrast, SEC 2D showed a highly variable effect on COL1A1 expression and a less pronounced effect on HAS2 compared to SEC 3D (Figure 9).

### 3.7. Effect of Secretome on Fibroblast Migration

**Figure 10:**
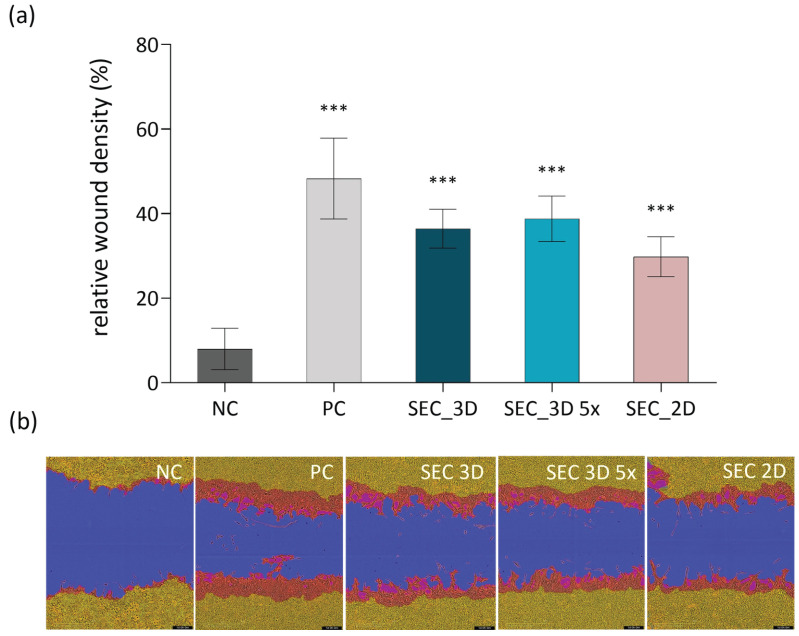
Effect of cASC SEC treatment on NIH 3T3 fibroblast migration. (**a**) Quantification of 3T3 cell density in the wound area after treatment with control (NC, growth medium without FBS), SEC 3D and concentrated SEC 3D 5×. All test conditions were performed in serum-free medium (0% FBS). The positive control (PC) consisted of growth medium supplemented with 10% FBS to stimulate cell migration. Each sample was performed in triplicate in three independent assays. Statistical analysis was performed using one-way ANOVA followed by Dunnett’s post hoc test. Results are presented as mean ± SD. Statistical significance was calculated for each sample in comparison to NC; *** *p ≤* 0.001. (**b**) Representative images from the scratch assay showing NIH 3T3 fibroblast migration 24 h post-wounding. Masks were applied using the Incucyte system to highlight the wound area (blue), initial scratch wound area (red) and migrating cells (yellow).

The effect of cASC secretome on fibroblast migration was evaluated using the scratch assay with NIH 3T3 cells (Figure 10a). Quantitative analysis demonstrated that both SEC 2D and SEC 3D significantly enhanced wound closure compared to the negative control. The effect of SEC 3D was more pronounced compared to SEC 2D, indicating a higher pro-migratory potential. Treatment with SEC 3D and SEC 3D 5× promoted comparable wound closure, with only a slight additional effect at higher concentration. Representative images at 24 h post-scratch (Figure 10b) support these findings, showing minimal migration in the control group, whereas SEC_3D and SEC_3D 5× promoted marked wound closure.

## 4. Discussion

In this study, we aimed to assess the immunomodulatory and pro-regenerative potential of the secretome derived from cASC cultivated under defined bioprocessing conditions. These conditions included 3D dynamic culture in a small-scale Applikon Minibio stirred tank bioreactor, exposure to hypoxic conditions (5% O_2_), and supplementation with 2% PLT. To the best of our knowledge, no study to date has directly evaluated the combined effects of hypoxic preconditioning, PLT supplementation, and 3D dynamic culture of cASC in a stirred tank bioreactor system. The stirred tank bioreactor offers a scalable and well-controlled environment that supports efficient 3D expansion of MSC, enhances paracrine signaling, and better mimics the native stem cell niche [4,30]. Building on this, microcarrier-based cultivation of cASC was established in a controlled stirred-tank bioreactor system. To optimize expansion efficiency, Cultispher S microcarriers were employed [23,31], due to their macroporous structure, which potentially provides protective niches for cells in stirred-tank bioreactors [23].

Importantly, cultivation under hypoxic conditions was applied in our setup. Hypoxia has been shown to significantly modulate the composition of the MSC secretome, particularly by increasing the secretion of anti-inflammatory mediators and angiogenic growth factors [32]. This effect is attributed to the activation of hypoxia-responsive signaling pathways, including HIF-1α and STAT-3, which promote a shift in MSCs towards a more pro-regenerative and immunomodulatory phenotype. In this context, HIF-1α has been demonstrated to enhance MSC survival and functional activity under hypoxic conditions, thereby reinforcing their therapeutic potential [33,34].

A comprehensive characterization of the cASC-derived secretome identified MCP1 as the most abundantly secreted chemokine, along with other factors including VEGFA, IL-6, and TGFβ [2]. VEGFA is a well-characterized pro-angiogenic factor, whereas MCP-1 is a chemokine that recruits inflammatory cells and modulates the expression of other inflammatory mediators, playing a key role in tissue remodeling and immune responses [35]. In our study, we observed that SEC 3D cultured in PLT-supplemented medium under hypoxic conditions, exhibited a significantly increased concentration of both VEGFA and MCP1 (Figure 6). In our study, the same cell concentration per milliliter of medium was used for both the 2D culture flasks and the 3D bioreactor system with microcarriers. Despite this, the 3D environment provides a more physiologically relevant spatial organization, enabling cells to grow in three dimensions, which likely increases local cell density and enhances cell–cell interactions. This improved microenvironment may contribute to the significantly higher secretion of pro-regenerative factors such as VEGFA and MCP1 observed in the 3D secretome. Thus, the enhanced paracrine activity in 3D culture may result from both changes in cellular phenotype and the effective increase in active cell mass within the bioreactor. The upregulation of VEGFA is consistent with previous findings in human [36] and pig ASC [27], where hypoxic preconditioning alone enhanced angiogenic factor secretion as well as with a recent study showing that VEGF-A and bFGF exhibited higher mRNA levels in 3D spheroid cultures compared to 2D cultures [37]. However, increased MCP1 secretion was not reported in those studies and no chemokine upregulation was observed in pig ASC under hypoxia [27]. A recent study on human MSCs similarly reported enhanced MCP1 expression under 3D spheroid culture [38], consistent with our observations. While this effect is therefore not unique in a broader MSC context, our work provides novel evidence for MCP1 upregulation in cASC under combined PLT supplementation, hypoxic preconditioning, and 3D bioreactor-based culture. Importantly, this chemokine may contribute to the regenerative potential of the secretome by orchestrating immune cell recruitment and promoting polarization of macrophages toward a pro-repair M2 phenotype [38,39,40]. Through these mechanisms, MCP-1 supports tissue regeneration, including angiogenesis and controlled modulation of inflammation.

The immunomodulatory effect of MSC has been demonstrated in numerous studies. In our study, the application of cASC 3D-derived secretome significantly reduced TNF-α expression in LPS-stimulated macrophages (Figure 7), suggesting its potential role in the modulation of macrophage-driven inflammatory responses. These data are consistent with findings reported by Heo et al. (2021) [28], where similar effects were observed. While most assays included both 2D- and 3D-derived secretomes, TNF-α expression in LPS-stimulated macrophages was evaluated only with the 3D secretome due to prioritization of experiments and limited sample availability. Nevertheless, the significant anti-inflammatory effect observed is consistent with previous reports demonstrating enhanced bioactivity of secretomes derived from 3D cultures. Specifically, secretome from 3D-cultured cASC spheroids significantly reduced the expression of TNFα, IL-1β, and IL-6 compared to secretomes from 2D cultures [37]. This effect was accompanied by a suppression of NF-κB activity (Figure 8), a key transcription factor regulating pro-inflammatory cytokine production. These results suggest that the anti-inflammatory potential of the secretome is mediated, at least in part, through modulation of the NF-κB signaling pathway in activated macrophages. Our observations are in line with previous studies reporting that MSC-secretome promotes a shift from pro-inflammatory M1 to anti-inflammatory M2 macrophage phenotypes, contributing to the resolution of inflammation [41]. A similar anti-inflammatory effect was reported by Xiao et al. (2020), where human MSC reversed LPS—induced epithelial–mesenchymal transition and lung injury by suppressing NF-κB and Hedgehog signaling pathways [42]. These results suggest that the immunomodulatory properties of MSC secretome are conserved across species and cellular contexts, and they reinforce the central role of NF-κB signaling in inflammation regulation. One possible mechanism by which the MSC secretome may suppress NF-κB signaling is via secreted anti-inflammatory cytokines such as IL-10 and TGF-β, which can stabilize IκB-α and inhibit its phosphorylation or degradation, preventing nuclear translocation of the p65/p50 complex [34,43]. In addition, in osteoarthritis models, MSC exosomes have been shown to inhibit phosphorylation of TGF-β-activated kinase 1 and IκBα, thus preventing NF-κB nuclear translocation and subsequent induction of pro-inflammatory cytokines [44,45].

Our results demonstrate that cASC secretome, produced under hypoxia and PLT-supplemented 3D culture, significantly reduced *COL1A1* and *HAS2* mRNA levels in TGF-β1-activated myofibroblasts (Figure 9), suggesting that 3D culture conditions may better preserve or even enhance the anti-fibrotic properties of the cASC-derived secretome. This aligns with previous findings showing MSC secretome’s ability to attenuate fibrotic responses by downregulating *COL1A1* via microRNA-mediated fibroblast modulation [46]. Consistently, Kanemoto et al. demonstrated that in canine chronic hepatitis, the expression levels of several fibrosis-related genes, including *COL1A1*, *COL3A1*, *TGF-β1*, *TGF-β2*, *PDGFB*, *PDGFD*, *TIMP1*, *MMP2*, and *THBS1*, correlated strongly with the histologic degree of fibrosis [47]. The decreased *HAS2* expression, a key enzyme in hyaluronan synthesis and fibrosis, further highlights the secretome’s regenerative and matrix-remodeling effects. Notably, HAS2 inhibition impairs myofibroblast activation and ECM deposition independently of Smad2/3 signaling [48]. In this context, it is important to note that platelet lysate itself contains a rich mixture of growth factors such as PDGF, IGF-1, EGF, FGF, and TGF-β, which can modulate fibroblast activity, ECM remodeling, and inflammatory signaling. These factors may therefore contribute synergistically to the observed downregulation of gene expression of *COL1A1* and *HAS2*, reinforcing the anti-fibrotic and regenerative potential of the cASC secretome [49,50].

Our findings confirmed that the secretome obtained from the optimized culture system significantly promoted fibroblast migration, as demonstrated by scratch assay analysis (Figure 10). This pro-migratory effect aligns with previous results from both canine and human MSC models, where secretome enhanced fibroblast motility through paracrine signaling mechanisms [51,52]. In particular, canine amniotic MSC-derived secretome was shown to stimulate fibroblast migration via key growth factors such as EGF, bFGF, and TGF-β, which are well-established regulators of cell proliferation and migration [51]. Moreover, MSC-derived secretome is known to contain a rich array of bioactive molecules such as MCP-1, TGF-β1, IL-6, VEGF, EGF, FGF, PDGF, and others, all of which contribute to the orchestration of fibroblast recruitment, migration, ECM deposition, and angiogenesis during the wound-repair process [53].

Furthermore, the use of a serum-free, PLT-supplemented medium in this study supports the trend toward xeno-free, good manufacturing practice (GMP)-compliant culture systems for the standardized production of therapeutic secretomes. PLT provides essential growth factors that enhance ASC viability and secretory function, while maintaining clinical safety [54,55]. In combination with dynamic 3D culture in a stirred-tank bioreactor and hypoxic preconditioning, this strategy appears to synergistically enhance the paracrine activity of ASC compared to 2D cultures under identical hypoxic and PLT-supplemented conditions. To our knowledge, this is one of the first demonstrations of cASC-derived secretome produced under such conditions, with measurable effects on molecular pathways associated with inflammation and tissue regeneration. Nevertheless, this study has several limitations. First, it was performed in a small-scale bioreactor system, and scalability to larger GMP-compliant settings remains to be validated. Second, the molecular mechanisms underlying the observed effects were not fully dissected, and more detailed proteomic and transcriptomic profiling will be necessary. Third, in vitro models provide only partial insight into therapeutic efficacy, and further in vivo validation is required to confirm clinical relevance. Additionally, a limitation of the present study is that the functional assays were performed using murine and human cell lines rather than canine cells. Species-specific differences in cytokine receptors, intracellular signaling, and paracrine responsiveness may influence the observed effects of the canine ASC secretome. While previous studies have demonstrated some degree of cross-species activity, confirmatory experiments using canine primary cells or commercially available canine cell lines would strengthen the translational relevance of the findings. Addressing this in future work will allow a more accurate characterization of the functional potential of the secretome in a species-appropriate context. Overcoming these limitations is crucial for translation to veterinary applications and, due to the close immunological and pathological parallels [56,57], may also support the development of human cell-free therapies.

## 5. Conclusions

This study demonstrates that culturing cASC in a stirred-tank bioreactor under hypoxia with PLT supplementation produces a secretome with enhanced biological activity compared to 2D cultures under the same conditions. The 3D-derived secretome showed improved anti-inflammatory, pro-migratory, and antifibrotic effects, highlighting the distinct advantages of combining dynamic 3D culture with hypoxic and PLT-enriched conditions for regenerative veterinary applications such as musculoskeletal injury repair, treatment of chronic wounds, and modulation of inflammatory or fibrotic disorders in veterinary patients.

## Figures and Tables

**Table 1:** Donor information for canine adipose tissue samples.

Breed	Sex	Age
Rottweiler	Female	8 years
Malamute	Female	Not available
Shih Tzu	Female	8 years
Border Collie	Female	3 years
Not available	Female	2 years
Labrador Retriever	Female	2 years
Border Collie	Female	1 year

## Data Availability

The original contributions presented in this study are included in the article. Further inquiries can be directed to the corresponding author.

## Abbreviations

The following abbreviations are used in this manuscript:

ASC	adipose-derived mesenchymal stem cells
cASC	canine adipose-derived mesenchymal stem cells
COL1A1	Collagen type I alpha I
COL3A1	collagen type III alpha 1
ECM	extracellular matrix
ELISA	enzyme-linked immuno sorbent assay
FBS	fetal bovine serum
EGF	epidermal growth factor
FGF	fibroblast growth factor
GMP	good manufacturing practice
HAS2	hyaluronan synthase 2
IACUC	institutional animal care and use committee
IGF-1	insulin-like growth factor 1
IL-6	Interleukin 6
IL-10	Interleukin 10
LPS	lipopolysaccharide
MCP-1	monocyte chemoattractant protein-1
MMP2	matrix metallopeptidase 2
MSC	mesenchymal stem cells
NF-κB	nuclear factor kappa B
PDGF	platelet-derived growth factor
PLT	platelet lysate
PMA	phorbol 12-myristate 13-acetate
SEC 2D	secretome collected from cASC cultured in 2D system
SEC 3D	secretome collected from cASC cultured in 3D system
SEC 3D 5×	secretomes collected from cASC cultured in 3D, 5× concentrated
TGF-β1	transforming growth factor beta-1
TGF-β2	transforming growth factor beta-2
THBS1	thrombospondin 1
TIMP1	tissue inhibitor of metalloproteinases 1
VEGF	vascular endothelial growth factor
